# Impact of Case and Control Selection on Training Artificial Intelligence Screening of Cardiac Amyloidosis

**DOI:** 10.1016/j.jacadv.2024.100998

**Published:** 2024-06-12

**Authors:** Amey Vrudhula, Lily Stern, Paul C. Cheng, Piero Ricchiuto, Chathuri Daluwatte, Ronald Witteles, Jignesh Patel, David Ouyang

**Affiliations:** a Department of Cardiology, Smidt Heart Institute, Cedars-Sinai Medical Center, Los Angeles, California, USA; b Division of Cardiology, Department of Medicine, Stanford University, Palo Alto, California, USA; c Alexion Pharmaceuticals, Boston, Massachusetts, USA; d Division of Artificial Intelligence in Medicine, Cedars-Sinai Medical Center, Los Angeles, California, USA

**Keywords:** artificial intelligence, cardiac amyloidosis, case selection, control selection, screening

## Abstract

**Background:**

Recent studies suggest that cardiac amyloidosis (CA) is significantly underdiagnosed. For rare diseases like CA, the optimal selection of cases and controls for artificial intelligence model training is unknown and can significantly impact model performance.

**Objectives:**

This study evaluates the performance of electrocardiogram (ECG) waveform-based artificial intelligence models for CA screening and assesses impact of different criteria for defining cases and controls.

**Methods:**

Using a primary cohort of ∼1.3 million ECGs from 341,989 patients, models were trained using different case and control definitions. Case definitions included ECGs from patients with an amyloidosis diagnosis by International Classification of Diseases-9/10 code, patients with CA, and patients seen in CA clinic. Models were then tested on test cohorts with identical selection criteria as well as a Cedars-Sinai general patient population cohort.

**Results:**

In matched held-out test data sets, different model AUCs ranged from 0.660 (95% CI: 0.642-0.736) to 0.898 (95% CI: 0.868-0.924). However, algorithms exhibited variable generalizability when tested on a Cedars-Sinai general patient population cohort, with AUCs dropping to 0.467 (95% CI: 0.443-0.491) to 0.898 (95% CI: 0.870-0.923). Models trained on more well-curated patient cases resulted in higher AUCs on similarly constructed test cohorts. However, all models performed similarly in the overall Cedars-Sinai general patient population cohort. A model trained with International Classification of Diseases 9/10 cases and population controls matched for age and sex resulted in the best screening performance.

**Conclusions:**

Models performed similarly in population screening, regardless of stringency of cases used during training, showing that institutions without dedicated amyloid clinics can train meaningful models on less curated CA cases. Additionally, AUC or other metrics alone are insufficient in evaluating deep learning algorithm performance. Instead, evaluation in the most clinically meaningful population is key.

Cardiac amyloidosis (CA), an underdiagnosed disease driven by myocardial deposition of misfolded amyloid protein, is a progressive condition responsible for substantial morbidity and mortality.^1^, ^2^, ^3^, ^4^ While early epidemiological data suggested a very low prevalence, autopsy series have found transthyretin amyloid (ATTR) deposits in 20 to 40% of octogenarians.^5^^,^^6^ CA has been reported to be the etiology in up to 13% of patients with heart failure with preserved ejection fraction, and ATTR CA is present in approximately 16% of patients with severe calcific aortic stenosis undergoing transcatheter aortic valve replacement.^7^, ^8^, ^9^ Newer data with more sensitive imaging suggest the prevalence might be as high as 1 to 2% of the general population.^10^ Regional disparities in CA diagnosis also exist and particularly impact Black Americans, who are disproportionately affected by the hereditary form of ATTR but are significantly undiagnosed.^11^

CA is challenging to diagnose because the disease is often indolent, and the symptoms are often similar to those of more common cardiac conditions. The disease is frequently not recognized until it has progressed to advanced stages, at which point treatment options are more limited. Timely diagnosis is vital, as new highly effective targeted therapies have been introduced for both light chain amyloid (AL) and ATTR CA, and these treatments display the greatest benefit when started early in the disease course.^12^, ^13^, ^14^ Previous work has demonstrated artificial intelligence’s (AI) ability to precisely phenotype diseases and characterize subtle cardiac physiology.^15^, ^16^, ^17^, ^18^, ^19^ Deep learning models have therefore been proposed to screen for CA using a variety of different forms of input data.^20^, ^21^, ^22^, ^23^ While such models have demonstrated strong performance, given the scarcity and underdiagnosis of CA, these models are often trained on limited data sets without external validation. Similarly, these case and control selection issues are not limited to amyloidosis and instead extend to other rare diseases, where the use of limited data sets and incomplete evaluation may also bottleneck clinical translatability of models. Further investigation into how cohort selection influences model performance is warranted, both for CA and other rare diseases.^22^, ^23^, ^24^, ^25^

In this study, we sought to evaluate the impact of case and control definitions in the training of an AI to identify CA. In doing so, we hoped to understand whether centers without dedicated amyloid clinics have the ability to train models using less stringent case definitions representing less curated cases of amyloidosis. We chose electrocardiogram (ECG) waveforms for model input as ECGs are inexpensive, noninvasive, widely available, and frequently obtained during routine visits. A variety of selection criteria as well as different ways to balance characteristics between cases and controls in the training data set were used to evaluate the impact of training design choices on AI model performance. By maintaining the same AI model architecture, type of input data, and site across the experiments, we sought to evaluate whether more or less stringent case and control definitions would impact expected population screening performance in a cohort of patients at a large, quaternary care center.

## Methods

### Data sources and study population

The study included ECGs from patients receiving care at Cedars-Sinai Medical Center between 2005 and 2022. The data were split 80% for training, 10% for internal validation, and 10% for testing on a patient level prior to model development; when multiple ECGs were present for a given patient, all were included in the same split, such that a patient in the training split had no ECGs in the validation or test splits, and vice versa. Similarly, to allow for accurate comparison between models trained with different case and control criteria, splits remained the same, such that all models developed, irrespective of inclusion and exclusion criteria, were trained on data from the train split and were evaluated on the held-out test split. All training cohorts were matched 1:10 on cases and controls coming from the training split. Models were evaluated on ECGs from the held-out test split, with cohorts that matched training cohort selection criteria as well as the entire test split (population prevalence). The entire test split (referred to as the Cedars-Sinai general patient population cohort) contained 1,130 ECGs from 105 patients with an International Classification of Diseases (ICD)-9/10 code of amyloidosis and 133,375 control ECGs from 34,089 patients. ECG waveform data, acquired at a sampling rate of 500 Hz, were extracted as 10 second, 12x5000 matrices of amplitude values. ECGs with missing leads were excluded from the study cohort. Associated clinical data for each patient, including demographic and clinical characteristics (eg, age, sex, body mass index, cardiovascular disease), were obtained from the electronic health record (EHR). Disease diagnoses were identified by ICD 9th and 10th edition codes, which were also obtained from the EHR. The Institutional Review Boards of Cedars-Sinai Medical Center and Stanford Healthcare approved the study protocol.

### CA AI model design and training

A convolutional neural network for ECG interpretation was designed to detect the presence of CA. The model architecture is similar to those previously described to evaluate postoperative outcomes and screen for chronic kidney disease.^26^^,^^27^ Briefly, the architecture was a lightly modified EfficientNet, consisting of atrous convolutions followed by multichannel 1D convolutions. The convolutional layers had an inverted residual structure, and their outputs were bottleneck layers with an intermediate expansion layer. In each set of expansion layers, the number of input channels increased to allow for information integration across the 12 leads of the ECG.^28^

The model was trained using the PyTorch Lightning deep learning framework to predict outcomes with the input of one 12-lead ECG. If the same patient had multiple ECGs, each ECG was considered an independent example during training. Models were initialized with random weights and trained using a binary cross entropy loss function for up to 100 epochs with an ADAM optimizer and an initial learning rate of 1e-2. Early stopping was performed based on the validation data set’s area under the receiver operating curve (AUC). The best model was determined based on screening performance in the Cedars-Sinai general patient population hold-out test cohort. This model was used for downstream analysis.

### Case and control definitions and test populations

Three progressively more selective definitions of amyloid were evaluated to understand whether more highly curated amyloid cases result in models with better screening performance or whether models trained with less curated cases can achieve similar performance. The broadest case definition used was a diagnosis of amyloidosis by ICD-9/10 code (n = 990). The second case definition was for CA, defined by a subset of patients from the first cohort but also having evidence of cardiac involvement (n = 686). Cardiac involvement was defined as having an abnormal interventricular septum (IVS) measurement, brain natriuretic peptide (BNP), or troponin within 180 days of ECG. 180 days was chosen given the indolent nature of amyloidosis development. The third case cohort were patients seen in cardiac amyloid clinic (n = 168) with documented diagnosis by biopsy or 99mTc-technetium pyrophosphate scintigraphy (PYP) scan after ruling out circulating monoclonal proteins by serum free light chains, as well as serum and urine immunofixation. Different populations of non-amyloid patients (defined as patients without an amyloidosis ICD-9/10 code) were chosen as controls. Control cohorts chosen for comparison include all non-amyloid patients, non-amyloid patients with left ventricular hypertrophy, non-amyloid patients with heart failure, and non-amyloid patients with heart failure with reduced ejection fraction (HFrEF). In various experimental setups, cases and controls were matched on different combinations of age, sex, wall thickness, and QRS amplitude to understand how these variables affected model performance. Wall thickness measurements were obtained from the closest echocardiogram within 180 days of the ECG. Case and control ratios were always 1:10 (as 1:1 and 1:100 ratios showed no significant difference in model performance, as shown in Supplemental Table 2), except for HFrEF, where a ratio of 1:4.5 was used as HFrEF cases were uncommon in the control set.

### Statistical analysis

A hold-out test data set which was never seen during model training was used to assess model performance. Model performance was assessed by testing on 3 different test cohorts: 1) a cohort mirroring the training and internal validation criteria in definitions and ratios of case and controls; 2) a Cedars-Sinai general patient population cohort with cases defined as patients with ICD-9/10 codes for amyloidosis (Supplemental Table 1); and 3) the Cedars-Sinai general patient population cohort with cases defined as amyloid clinic patients. The Cedars-Sinai general patient population cohort consisted of all ECGs in 10% patient-level test split of the primary cohort of 1,344,372 ECGs. Meanwhile, the first test cohort was generated using ECGs from the Cedars-Sinai general patient population cohort with the same case and control criteria and ratio found in the training and internal validation sets for each model. The best model was determined based on population-level screening performance in identifying cases in the Cedars-Sinai general patient population cohort. Model performance in identifying CA was assessed via AUC. Two-sided 95% confidence intervals were computed using 10,000 bootstrapped samples for each metric. Statistical analysis was performed in R, version 4.2.2 (R Project for Statistical Computing) and Python, version 3.7.0 (Python Software Foundation).

### Wall thickness AI model design and training

The secondary model trained to predict wall thickness was trained using the same modified Efficient-Net architecture mentioned above. However, given that the model predicted wall thickness (a regression task), mean squared error loss was employed during model training. Similarly, to maximize number of training examples, all ECGs at Cedars-Sinai Medical Center associated with an echocardiogram within 180 days were used to train the wall thickness model. This was more than the number of ECGs (cases and controls) used to train CA models. Similarly, while the majority of cases and controls for CA models had an echo within 180 days of ECG, some did not. Therefore, the same training and test splits as the CA models were not used in evaluating the wall thickness model.

## Results

### Population characteristics

Our primary cohort consisted of 1,344,372 ECGs from 341,989 patients at Cedars-Sinai Medical Center. Amyloidosis cases comprised 10,042 ECGs across 990 patients, with CA representing 7,507 ECGs across 686 patients, and clinic patients represented by 2,256 ECGs from 168 patients. Demographics, comorbidities, and ECG characteristics are detailed in Table 1. Compared to controls, amyloid cases had a higher proportion of males, a higher proportion of Black individuals, and the average age was older.

**Table 1:** Case and Control Demographics

	Amyloid by ICD-9/10	Cardiac Amyloidosis	Curated Cardiac Amyloidosis Clinic	Controls
Number of ECGs	10,042	7,507	2,256	1,334,330
N	990	686	168	340,999
Male	70.17% (7,046)	72.97% (5,478)	85.55% (1,930)	53.78% (715,999)
Age (y)	70.62 ± 11.92	70.44 ± 11.94	70.26 ± 9.68	63.83 ± 18.87
Black race	23.76% (2,380)	25.35% (1,902)	23.96% (540)	16.47% (218,049)
Hypertension	48.46% (4,866)	50.66% (3,803)	45.26% (1,021)	31.46% (419,750)
Diabetes mellitus	23.02% (2,312)	24.51% (1,840)	28.65% (632)	15.57% (207,725)
Coronary artery disease	45.32% (4,552)	48.39% (3,633)	43.34% (956)	24.51% (327,050)
Heart failure/cardiomyopathy	36.99% (3,715)	27.00% (2,027)	16.18% (357)	21.80% (290,898)
IVS (cm)	1.30 (1.08-1.57)	1.33 (1.10-1.60)	1.36 (1.10-1.70)	1.10 (0.93-1.28)
LVPW (cm)	1.27 (1.06-1.53)	1.30 (1.10-1.58)	1.32 (1.10-1.68)	1.09 (0.93-1.21)
IVS or LVPW >1.2 cm^a^	61.74% (4,875)	68.27% (4,546)	66.43% (1,320)	34.98% (239,161)
ECG amplitude (mV)	0.633 ± 0.295	0.632 ± 0.310	0.614 ± 0.390	0.695 ± 0.618
Heart rate (beats/min)	81.63 ± 21.83	83.65 ± 22.14	86.25 ± 21.51	80.89 ± 21.90
Abnormal ECG	63.92% (6,418)	68.03% (5,107)	61.92% (1,397)	51.86% (691,990)
Bundle branch block	17.71% (1,779)	18.90% (1,419)	19.24% (434)	12.18% (162,481)
Ischemia	13.02% (1,307)	14.80% (1,111)	11.44% (258)	10.06% (134,230)
Infarct	29.74% (2,986)	32.86% (2,467)	29.48% (665)	17.79% (237,326)
Right axis deviation	1.30% (131)	1.48% (111)	1.51% (34)	0.69% (9243)
Left axis deviation	17.21% (1,728)	18.25% (1,370)	17.33% (391)	9.393% (125,334)

Values are n, % (n), mean ± SD, or median (IQR).

bpm = beats per minute; ECG = electrocardiogram; ICD = International Classification of Diseases; IVS = interventricular septum; LVPW = left ventricular posterior wall; mV = millivolts.

a Averages, counts, and percentages for these variables are based on the total number of patients with nearest laboratory testing or echocardiogram study within 180 days.

### Model performance in defined cohort and cedars-sinai general patient population

Models trained on CA by ICD-9/10 code definition and on ECGs from patients seen in CA clinic achieved similar performance (Table 2, Central Illustration). The best model used cases with CA (ICD-9/10 for amyloidosis with evidence of cardiac involvement: BNP >332, troponin >0.04, or left ventricular hypertrophy [LVH]) and controls matched by age and sex for model training. After optimizing case and control selection criteria and matching, our final model identified CA with an AUC of 0.820 (95% CI: 0.782-0.857) in a held-out test cohort where case and controls mimicked the training cohort and an AUC of 0.744 (95% CI: 0.721-0.767) in the Cedars-Sinai general patient population test cohort.

**Table 2:** Model Performance in Defined Cohort and Cedars-Sinai General Patient Population

					Population	
	Cases Criteria	Controls Criteria	Matching	Matched Cohort	Cases by ICD-9/10	Cases by Clinic List
Varying case definition	Amyloid by ICD-9/10^a^	Nonamyloid^d^	None	0.705 (0.679-0.730)	0.702 (0.677-0.727)	0.844 (0.803-0.88)
	Amyloid by ICD-9/10 with cardiac involvement^b^	Nonamyloid^d^	None	0.750 (0.726-0.773)	0.728 (0.706-0.751)	0.866 (0.830-0.898)
	Curated amyloid clinic list^c^	Nonamyloid^d^	None	0.880 (0.844-0.912)	0.720 (0.695-0.744)	0.880 (0.844-0.911)
Varying control definition	Amyloid by ICD-9/10^a^	Nonamyloid HFrEF^e^	None	0.767 (0.745-0.789)	0.467 (0.443-0.491)	0.426 (0.378-0.476)
	Amyloid by ICD-9/10^a^	Nonamyloid heart failure^f^	None	0.650 (0.625-0.674)	0.517 (0.491-0.544)	0.545 (0.494-0.606)
	Amyloid by ICD-9/10^a^	Nonamyloid LVH^g^	None	0.660 (0.642-0.736)	0.570 (0.545-0.596)	0.690 (0.642-0.736)
Varying case to control matching	Amyloid by ICD-9/10^a^	Nonamyloid^d^	Age and sex	0.682 (0.656-0.708)	0.698 (0.674-0.722)	0.861 (0.828-0.892)
	Amyloid by ICD-9/10^a^	Nonamyloid^d^	Age, sex, and wall thickness	0.662 (0.633-0.691)	0.659 (0.633-0.685)	0.819 (0.778-0.856)
	Amyloid by ICD-9/10^a^	Nonamyloid^d^	Age, sex, and QRS amplitude	0.677 (0.633-0.691)	0.636 (0.609-0.662)	0.775 (0.730-0.818)
Best models	Amyloid by ICD-9/10 with cardiac involvement^b^	Nonamyloid^d^	Age and sex	0.744 (0.721-0.767)	0.733 (0.711-0.754)	0.820 (0.782-0.857)
	Curated amyloid clinic list^c^	Nonamyloid	Age and sex	0.898 (0.868-0.924)	0.714 (0.690-0.738)	0.898 (0.870-0.923)

Values are AUC (95% CI).

HFrEF = heart failure with reduced ejection fraction; ICD = International Classification of Diseases.

a Cases included ECGs from patients with any ICD-9/10 code for amyloidosis.

b Cases included ECGs from patients with any ICD-9/10 code for amyloidosis AND with evidence of cardiac involvement within 180 days of an ICD code for cardiac involvement.

c Cases included ECGs from patients receiving care at the cardiac amyloidosis clinic at Cedars Sinai.

d Nonamyloid controls included ECGs from patients with no diagnosis of amyloidosis (no ICD-9/10 code for amyloidosis).

e Controls included ECGs from patients with HFrEF and no diagnosis of amyloidosis (no ICD-9/10 code for amyloidosis).

f Controls included ECGs from patients with heart failure but no diagnosis of amyloidosis (no ICD-9/10 code for amyloidosis).

g Controls included ECGs from patients with LVH without amyloidosis (no ICD-9/10 code for amyloidosis).

**Central Illustration:**
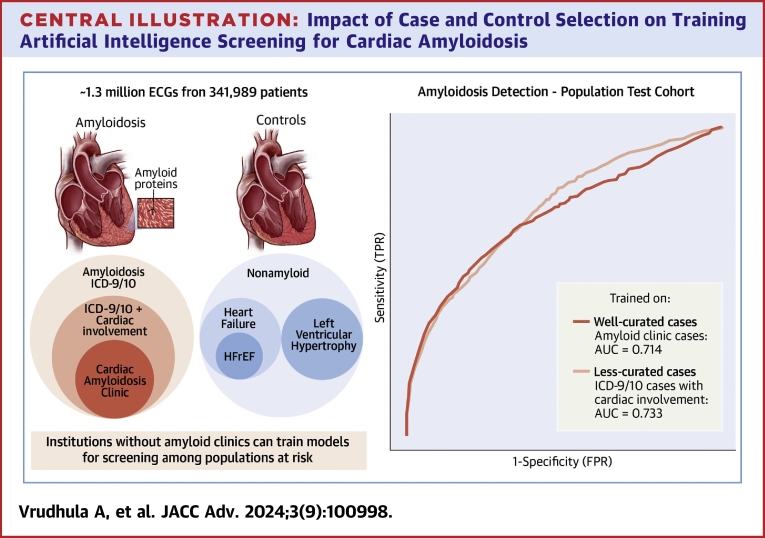
**Impact of Case and Control Selection on Training Artificial Intelligence Screening for Cardiac Amyloidosis** Model performance was evaluated while training with different case and control definitions. these results show that models trained on less curated cases perform just as well, if not better, on a population level than highly curated cases. This opens the door for centers without dedicated amyloid clinics to train models that could potentially be used as population screening tools. ECG = electrocardiogram; HFrEF = heart failure with reduced ejection fraction; ICD = International Classification of Diseases.

### Performance of varying case and control

When varying case definition, we found that stricter case inclusion criteria, namely: 1) CA (ICD-9/10 of amyloidosis with evidence of cardiac involvement [defined as LVH, BNP >332, or troponin >0.04]); and 2) clinic cases, resulted in increased AUC when tested on the matched held-out test set (AUC increasing from 0.705 to 0.880 with increased stringency). However, the improved performance did not generalize when tested on the population cohort (AUCs ranging from 0.702 to 0.728 with overlapping confidence intervals). Models in which cases and controls were matched for QRS amplitude performed the poorest of these matching combinations when tested on the Cedars-Sinai general patient population cohort for both CA ICD definitions and clinic cases definitions.

Models trained against controls that were phenotypically most distinct from amyloid resulted in the highest AUC in the matched test cohorts (for example, with HFrEF controls, the AUC was 0.767 (95% CI: 0.745-0.789), while controls with greater overlap with amyloid demonstrated lower AUCs in the matched cohorts (non-amyloid LVH controls resulted in an AUC of 0.660 (95% CI: 0.642-0.736). However, these results on matched cohorts did not correlate with AUCs in the Cedars-Sinai general patient population test cohort (Table 1), particularly in models in which the inclusion criteria for controls during testing were significantly different from those of the Cedars-Sinai general patient population test cohort. For all models, the choice of case definition in the held-out Cedars-Sinai general patient population test cohort significantly impacted AUC, as the AUC was consistently higher when cases were defined by clinic adjudication rather than by ICD9/10 definition (mean difference of 0.116 (0.068) across models). For downstream analyses, we chose the ICD9/10 definition of cases for evaluation of sensitivity/specificity as this difference in performance was likely due to later and more obvious phenotypes being seen in the clinic cohort; as we envision the use of such an algorithm for screening, the goal is to identify earlier cases.

### Secondary analyses

We investigated the utility of the model as a potential screening tool by measuring sensitivity, specificity, and predictive values at various screening thresholds (Table 3). At the Youden index, the model showed a sensitivity of 0.609 (95% CI: 0.569-0.648) and specificity of 0.718 (95% CI: 0.714-0.721). Positive predictive value was 0.018 (95% CI: 0.016-0.020) while negative predictive value was 0.995 (95% CI: 0.995-0.996). With a chosen specificity of 0.974, an estimated additional 275 patients would be identified out of every 10,000 patients screened, of which 18 patients would have amyloidosis. These 275 patients identified by the model could then undergo echocardiographic testing, where potential more sensitive modes of identifying amyloidosis have been developed.^20^ For high suspicion patients and after clinician evaluation, more expensive but specific methods of diagnosis like magnetic resonance imaging and PYP scans undertaken for downstream testing. The variation in positive predictive value was assessed at different algorithm detection thresholds and prevalence of amyloidosis. (Supplemental Table 3). Several populations are at higher risk for amyloidosis, and we examined model performance in these high-risk groups (Table 4). The AUC for males (0.756 [95% CI: 0.731-0.781]) and individuals >60 years of age (0.739 [95% CI: 0.716-0.763]) were consistently high.

**Table 3:** Assessing Model Utility As a Potential Screening Tool at Different Thresholds

Threshold	Sensitivity	Specificity	PPV	NPV	Positives per 10,000 Screened
0.05	0.934 (0.912-0.953)	0.233 (0.23-0.236)	0.010 (0.009-0.011)	0.998 (0.997-0.998)	7,682 (7651-7714)
0.101 (Youden index)	0.609 (0.569-0.648)	0.718 (0.714-0.721)	0.018 (0.016-0.020)	0.995 (0.995-0.996)	2,853 (2,818-2,887)
0.25	0.212 (0.178-0.246)	0.974 (0.973-0.975)	0.064 (0.053-0.076)	0.993 (0.993-0.994)	275 (263-288)
0.5	0.076 (0.056-0.100)	0.997 (0.996-0.997)	0.162 (0.120-0.207)	0.992 (0.992-0.993)	40 (35-45)
0.7	0.023 (0.011-0.036)	0.999 (0.999-1.000)	0.241 (0.132-0.361)	0.992 (0.991-0.992)	8 (6-10)

Values are AUC (95% CI).

NPV = negative predictive value; PPV = positive predictive value.

**Table 4:** Assessing Model Performance in Population Subsets

Population Subset	N	Similar Cohort	N	Population	Population Sensitivity^a^	Population Specificity^a^
All ECGs	10,648	0.744 (0.721-0.767)	134,845	0.733 (0.712-0.755)	0.609 (0.569-0.649)	0.718 (0.714-0.721)
Male	7,150	0.760 (0.733-0.787)	71,476	0.756 (0.731-0.781)	0.669 (0.622-0.716)	0.691 (0.686-0.696)
Age ≥60 y	8,536	0.764 (0.735-0.784)	84,648	0.739 (0.716-0.763)	0.651 (0.606-0.695)	0.686 (0.682-0.690)
African American race	1,801	0.664 (0.622-0.705)	21,930	0.681 (0.645-0.715)	0.491 (0.420-0.560)	0.718 (0.709-0.726)
LVH documented by echo	899	0.581 (0.525-0.633)	39,755	0.696 (0.668-0.723)	0.674 (0.627-0.718)	0.578 (0.571-0.585)
Normal heart rate	7,767	0.741 (0.714-0.767)	96,722	0.737 (0.712-0.761)	0.600 (0.549-0.642)	0.733 (0.729-0.737)
Heart failure or cardiomyopathy	3,146	0.748 (0.693-0.801)	38,908	0.754 (0.710-0.796)	0.633 (0.556-0.709)	0.714 (0.708-0.720)

Values are AUC (95% CI).

a Sensitivity and specificity were calculated using the Youden index as the threshold.

**Figure 1:**
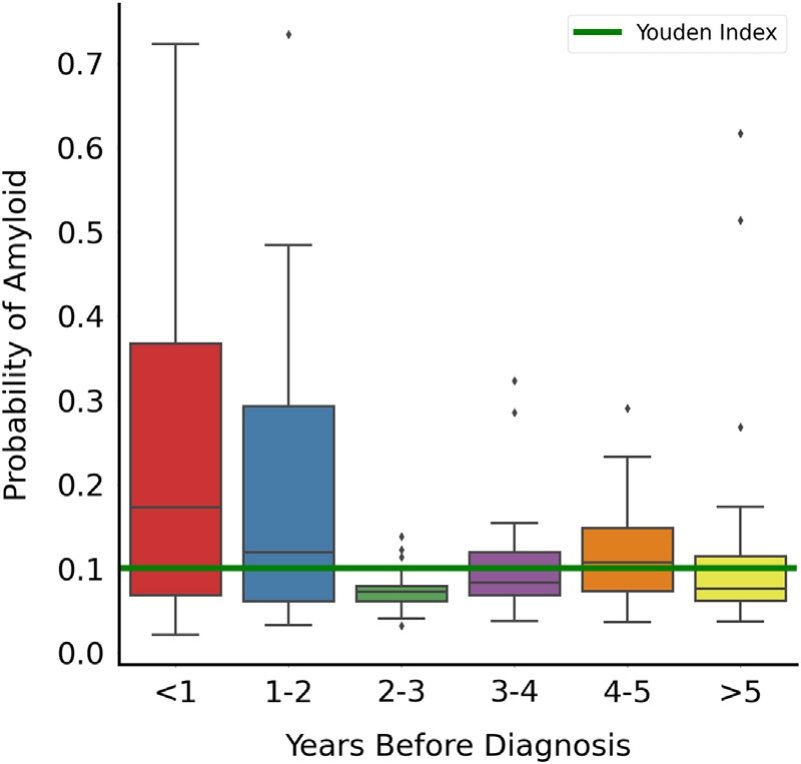
**Predicted Amyloid Probability Before Diagnosis** Probability of amyloid (y-axis) is shown here for ecgs taken before diagnosis to assess if the model can detect amyloidosis before date of clinical diagnosis. ECG = electrocardiogram.

**Figure 2:**
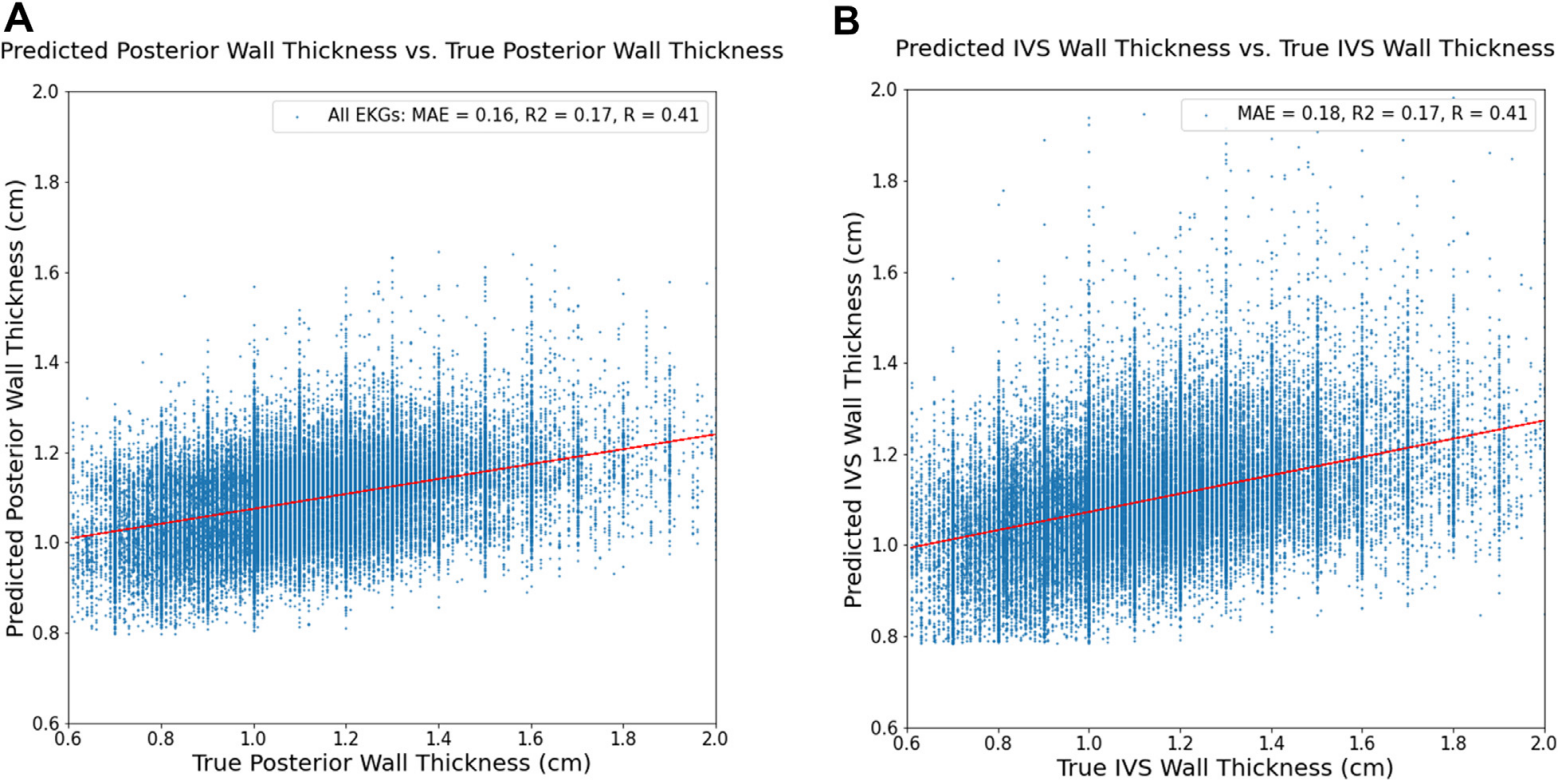
**Deep Learning Based Prediction of Left Ventricular Wall Thickness** (A) Predicted LVPW thickness. Prediction of left ventricular posterior wall (LVPW) thickness from ECGs using a deep learning model. (B) Predicted IVS thickness prediction of interventricular septal (IVS) thickness from ECGs using a deep learning model. ECG = electrocardiogram; MAE = mean absolute error.

Given that identifying CA early is paramount to reaping the greatest benefit from new therapies, we sought to gauge the model’s ability to predict CA prior to diagnosis. Detailed model predictions of ECGs before clinical diagnosis date are shown in Figure 1. Overall, at the Youden index, the model detected disease in a significant proportion of ECGs at least 2 years before first diagnosis. With the strong relationship between QRS amplitude and LVH, ECGs carry information that can be leveraged to predict wall thickness. We show that deep learning can predict wall thickness with a mean absolute error of 2 mm (Figure 2). The mean difference between true and predicted values for IVS and left ventricular posterior wall (LVPW) thickness models were 0.031 ± 0.237 and 0.011 ± 0.210. The relationship between the difference and mean of predicted and true wall thickness is shown in Supplemental Figures 4A and 4B. Similarly, the IVS and LVPW thickness models demonstrated AUCs of 0.727 (95% CI: 0.719-0.734) and 0.738 (95% CI: 0.729-0.747), respectively, for predicting wall thickness >1.5 cm. However, it should be noted that matching based on wall thickness measurements from echocardiograms or LVH (wall thickness >1.2 cm) did not improve screening performance of the model in the Cedars-Sinai general patient population test cohort, and similarly wall thickness alone was insufficient to identify CA.

## Discussion

As CA is a historically rare disease, AI screening has been limited by having few well-curated cases for model training. In this study, we reaffirm that CA can be identified in ECGs through AI evaluation.^22^^,^^23^ However, we also show that models perform similarly, regardless of being trained on highly curated clinic cases or less curated cases (based on ICD 9/10 codes). These findings open the door for community hospitals, Veterans Affairs centers, and other institutions without specific amyloid clinics to refine or train models for screening among populations at risk. In all models (regardless of how well curated training cases are), testing with clinic-derived lists of CA cases results in a higher AUC, likely due to the over-representation of later and more fulminant cases. However, in the Cedars-Sinai general patient population test cohort, models perform similarly in identifying patients with CA, regardless of the cases they were trained with. In the case of CA, clinic cohorts are likely enriched for patients with more severe disease, including those being considered for advanced therapies. These patients are likely easier to identify via AI detection but are not the optimal patients to identify via screening. These cases might not be representative of subtle early cases which one hopes to identify in disease screening and the model performance might be overestimated as more severe cases are easier to identify.

For this reason, we advocate that AUC or any standard performance metric alone (sensitivity, specificity, F1 score, precision, etc) without consideration of case/control selection is imperfect in benchmarking AI performance for CA and other rare diseases. No single quantitative metric solves this problem, but training and testing cohort composition should be considered in tandem in when assessing performance and clinical impact of AI models for CA and other rare diseases. While published AUCs on late-stage disease are higher, the best model here still shows potential in detecting CA. It remains unclear whether the current model possesses sufficient accuracy to serve as a CA screening tool. However, when compared to several clinically used screening tests (including those recommended by the United States Preventive Services Task Force), the results are encouraging. Sensitivities and specificities of Pap smear, monofilament testing for diabetic neuropathy, mammography, digital rectal exam, and serum prostate-specific antigen ranged from 0.65 to 0.85 and 0.79 to 0.96, respectively. The sensitivity and specificity of the best model at the Youden index were close to both of these ranges.^29^

Finally, we caution readers that choosing training cohorts for AI models mirrors designing a case-control study. Small choices in selection criteria can significantly impact model generalizability as models can identify shortcut variables and confounding influences. For example, as seen in Table 2, age and sex matching led to a model that outperformed one trained on unmatched cases and controls, presumably as matching cases and controls on age and sex forced the model to learn features outside of age and sex. Age and sex could be confounders that a model was learning to use as proxies to differentiate cases and controls. Meanwhile, matching on age, sex, and QRS amplitude generalized poorly. This makes intuitive sense as low ECG amplitude is a hallmark feature of amyloidosis. Elimination of any difference in QRS amplitude between cases and controls would force the model to rely on other features to distinguish cases and controls (pseudo-infarct patterns, etc) which are rarer and may not be seen in all cases when testing on a population level.

A truly generalizable model needs to be tested in external validation; however, given the difficulty of finding well-curated cohorts, in this study, we were unable to perform external validation. The difficulty in finding gold standard cohorts is inspiration of this work is to evaluate whether less well-curated cohorts can similarly be used to derive AI. A variety of screening algorithms have been proposed given the mortality benefit from early CA treatment, which include both EHR elements, echocardiogram interpretation, and ECG screening as well as physician prompting.^20^^,^^21^^,^^23^^,^^30^ All such approaches rely on referral to clinical experts, as even downstream testing with cardiac magnetic resonance imaging or PYP scans have imperfect test characteristics. Such approaches are difficult to model given uncertainty around the true incidence of CA in the population as well as misleading metrics given significant class imbalance. Additionally, implementation of AI algorithms is still rare, so further study must be undertaken to understand technical limitations and acceptability to physicians and patients.

### Study Limitations

First, there are limitations to disease definitions by ICD9/10 codes. While we show similar results with our curated clinic cohort, many patients with amyloidosis by ICD-9/10 codes do not have confirmatory testing available for review in the EHR. We did note that of the 168 well-curated amyloid clinic patients, 165 (98.2%) had an ICD-9/10 code for amyloidosis, suggesting high sensitivity of the ICD9 codes for CA. Additionally, this resulted in models that showed strong performance in cross-validation, suggesting patient similarity. In the current training and testing population, patients with ICD 9/10 codes for amyloidosis appear to have similar clinical characteristics as well-curated CA cases, given that models trained on less curated cases still performed well in detecting well-curated cases, rather than simply detecting which ECGs were associated with ICD codes. Similarly, wall thickness and QRS amplitude (Table 1) in the ICD cohort were similar to the CA clinic cohort.

Second, our selection of cardiac involvement requires selected laboratory testing or echocardiographic assessment to be done within 180 days, which might bias toward more severe or obvious cases of CA. While this is similarly true for patients in amyloid clinic, this degree of label noise might both result in false positives and false negatives. Further work is required to bring these findings to the bedside. Importantly, given the underdiagnosis of amyloidosis, there are likely patients in the control cohort who have undiagnosed CA, limiting the potential model performance. Additionally, most models for CA screening are restricted to a few centers, so validating this model at other centers is key to understanding the generalizability of these findings. It is important to recognize that the predictive value of ICD codes may be different at different institutions. However, the analyses presented, namely: 1) assessing performance of models trained on less curated cases in detecting well-curated amyloid cases; and 2) clinical characteristics based on ECG characteristics and clinical cardiac characteristics provide a starting point for researchers to understand the predictive value of ICD 9/10 codes for amyloidosis at their institutions when training models.

External validation was not included in the current study given that CA is a rare disease, and labels from an outside institution were not available to externally validate the model. Finally, while Figure 1 shows some promise regarding early detection, this does not provide conclusive evidence, as there is overlap with the Youden index even multiple years prior to a diagnosis date. A truly prospective study is necessary to gauge the true clinical impact of these AI models. Finally, given possible imperfect model calibration, probabilities yielded by models may not directly equal true probability for amyloidosis and would likely require thresholding at different operating points to achieve desired performance.

## Conclusions

CA is an underdiagnosed progressive disease with phenotypic heterogeneity, and care should be taken in understanding how AI models are trained to screen for CA. In this study, we found selection of cases and controls significantly impacted model performance on a Cedars-Sinai general patient population test cohort, with even a less curated case definition being able to train AI models. AUC alone is insufficient to assess the generalizability of these AI models, and further external validation as well as prospective validation is needed to understand the utility of screening AI models.

PERSPECTIVES **COMPETENCY IN MEDICAL KNOWLEDGE:** Selection of cases and controls can significantly impact the performance of deep learning models constructed to screen for CA. Case and controls selection, therefore, must be considered when evaluating deep learning models, as AUC and other standard metrics alone provide an incomplete picture. Instead, models should be evaluated in the clinically most meaningful population with consideration of cases and controls.

**TRANSLATIONAL OUTLOOK 1:** Models trained on less curated amyloidosis cases perform comparably to those trained on well-curated cases on population-level cohorts. These data open the door for centers without dedicated amyloid clinics to train models based on ICD 9/10 codes.

**TRANSLATIONAL OUTLOOK 2:** Prospective studies and multi-institutional validation are needed to understand model utility and bring models to the bedside.

## Funding support and author disclosures

At the time of this work, Dr Vrudhula was a research fellow supported by the Sarnoff Cardiovascular Research Fellowship. Drs Ricchiuto and Daluwatte are employees of Alexion Pharmaceuticals. All other authors have reported that they have no relationships relevant to the contents of this paper to disclose.
