# Supplementary material for: Impact of Case and Control Selection on Training Artificial Intelligence Screening of Cardiac Amyloidosis
Source: JACC Adv. 2024 Jun 12;3(9):100998. doi: 10.1016/j.jacadv.2024.100998 (PMC11450940; doi:10.1016/j.jacadv.2024.100998)
Supplement: Supplemental Data [file mmc1.pdf]

Supplemental Table 1 - ICD Codes

Code	***Name***	***Code Type***
277.3	*Amyloidosis*	*ICD 9*
277.30	*Amyloidosis*	*ICD 9*
277.39	*Other amyloidosis*	*ICD 9*
E85	*Amyloidosis*	*ICD 10*
E85.0	*Non-neuropathic heredofamilial amyloidosis*	*ICD 10*
E85.1	*Neuropathic heredofamilial amyloidosis*	*ICD 10*
E85.2	*Heredofamilial amyloidosis*	*ICD 10*
E85.4	*Organ-limited amyloidosis*	*ICD 10*
E85.8	*Other-limited amyloidosis*	*ICD 10*
E85.81	*Light chain (AL) amyloidosis*	*ICD 10*
E85.82	*Wild-type transthyretin-related (ATTR) amyloidosis*	*ICD 10*
E85.89	*Other amyloidosis*	*ICD 10*
E85.9	*Amyloidosis*	*ICD 10*

Supplemental Table 2: Model Performance while Varying Case: Control Ratios

Ratio Of Cases: Controls	Test Cohort	AUC
**1:1**	**CSGPP Test Cohort**	0.721 (0.699 -0.744)
	**CSGPP Test Cohort with Only Amyloid Clinic Cases**	0.864 (0.830 – 0.896)
	**Matched Test Cohort**	0.720 (0.688 – 0.751)
**1:10**	**CSGPP Test Cohort**	0.733 (0.711 – 0.754)
	**CSGPP Test Cohort with Only Amyloid Clinic Cases**	0.820 (0.782 – 0.857)
	**Matched Test Cohort**	0.744 (0.721 – 0.767)
**1:100**	**CSGPP Test Cohort**	0.694 (0.669 – 0.718)
	**CSGPP Test Cohort with Only Amyloid Clinic Cases**	0.842 (0.807 – 0.874)
	**Matched Test Cohort**	0.722 (0.693 – 0.749)

CSGPP Test Cohort = Cedars Sinai General Patient Population Test Cohort. The best model (trained using cardiac amyloidosis cases with age and sex matched controls), was retrained with different ratios of cases: controls). AUC on the CSGPP showed minimal change with the other ratios and was arguably the best for training when the ratio of cases to controls was 1:10.

Supplemental Table 3

Threshold	PPV @ 0.84 % Prevalence	PPV @ 5% Prevalence	PPV @ 10% Prevalence	PPV @ 20% Prevalence
**0.05**	0.010 (0.009 - 0.011)	0.060 (0.057-0.064)	0.118 (0.111-0.125)	0.234 (0.221-0.246)
**0.101** (Youden Index)	0.018 (0.016 - 0.020)	0.103 (0.096-0.111)	0.196 (0.182-0.209)	0.340 (0.320-0.361)
**0.25**	0.064 (0.053 - 0.076)	0.306 (0.058-0.325)	0.460 (0.417 - 0.504)	0.675 (0.626-0.724)
**0.5**	0.162 (0.120 - 0.207)	0.588 (0.507-0.667)	0.725 (0.645-0.808)	0.837 (0.766-0.907)
**0.7**	0.241 (0.132 - 0.361)	0.722 (0.571-0.875)	0.897 (0.786-1.00)	0.929 (0.833-1.0)

Supplemental Figure 2 – Precision Recall Curves and Model Calibration Curves: Precision Recall Curves for Models trained on with Different Case and Control Definitions are Shown Here

CSGPP Test Cohort = Cedars Sinai General Patient Population Test Cohort

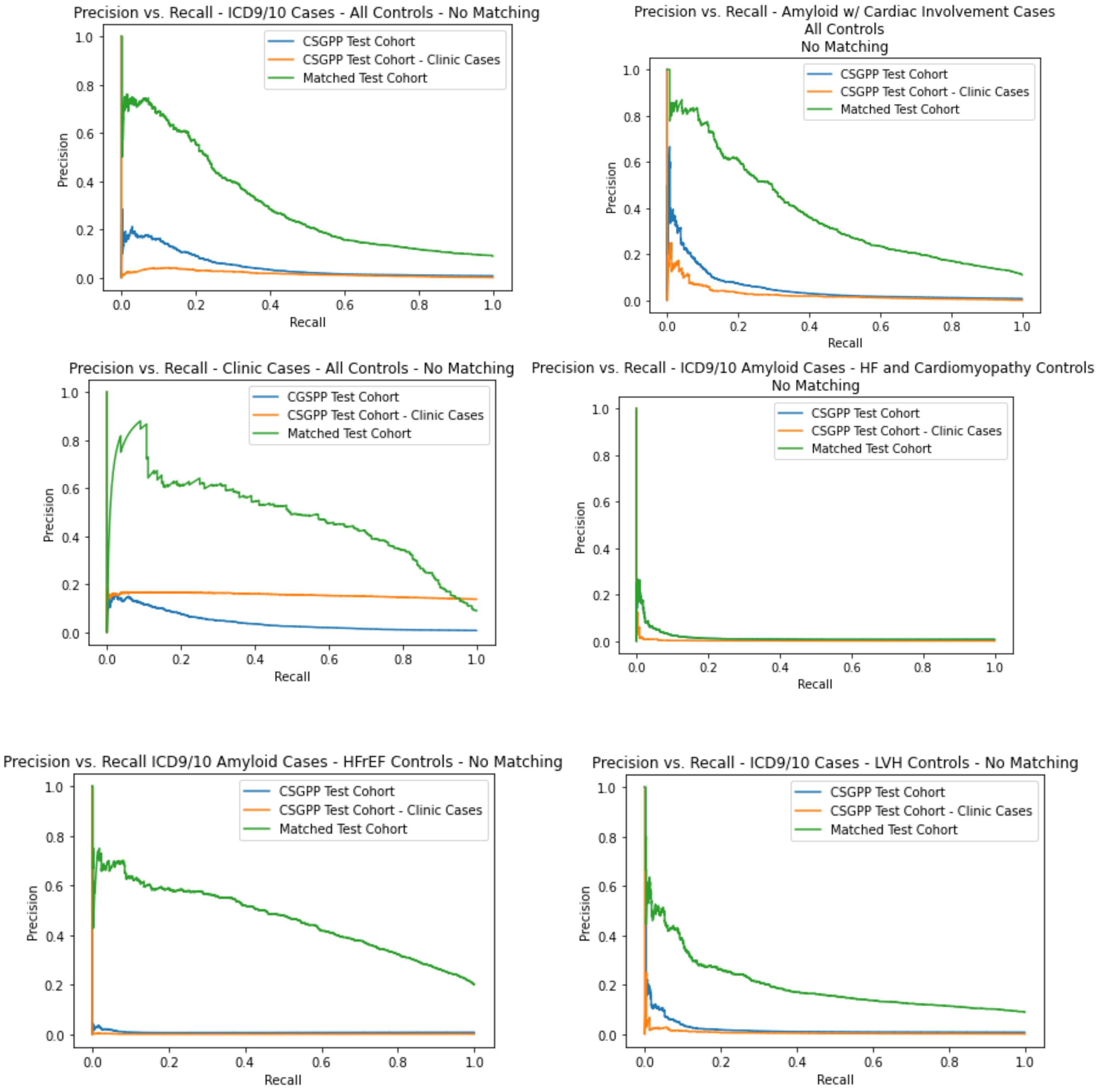

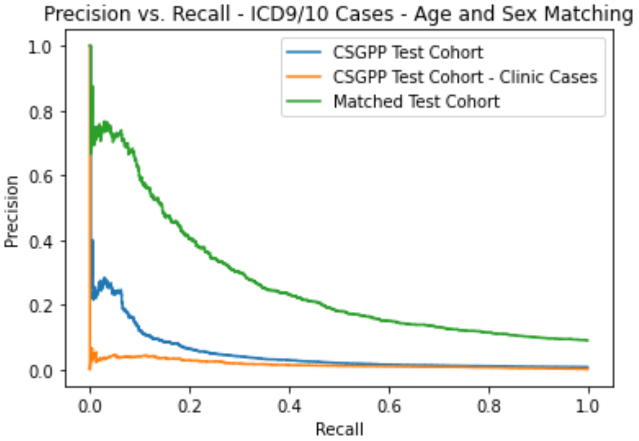
Precision vs. Recall - ICD9/10 Cases - Age and Sex Matching
CSGPP Test Cohort
CSGPP Test Cohort - Clinic Cases
Matched Test Cohort
Precision
Recall
0.0
0.2
0.4
0.6
0.8
1.0

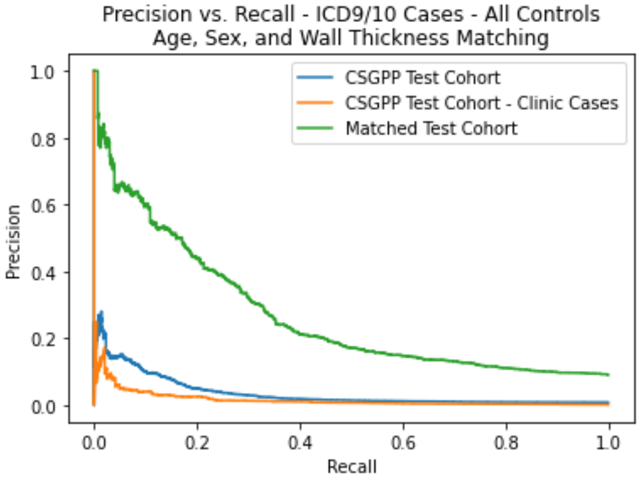
Precision vs. Recall - ICD9/10 Cases - All Controls
Age, Sex, and Wall Thickness Matching
CSGPP Test Cohort
CSGPP Test Cohort - Clinic Cases
Matched Test Cohort
Precision
Recall
0.0
0.2
0.4
0.6
0.8
1.0

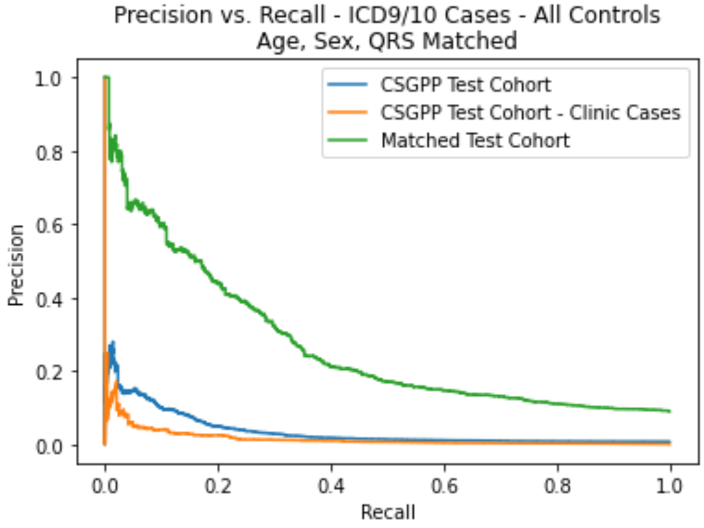
Precision vs. Recall - ICD9/10 Cases - All Controls
Age, Sex, QRS Matched
CSGPP Test Cohort
CSGPP Test Cohort - Clinic Cases
Matched Test Cohort
Precision
Recall
0.0
0.2
0.4
0.6
0.8
1.0

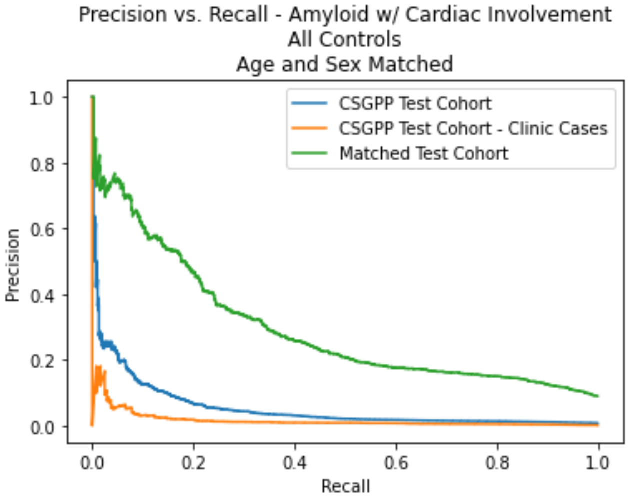
Precision vs. Recall - Amyloid w/ Cardiac Involvement
All Controls
Age and Sex Matched
CSGPP Test Cohort
CSGPP Test Cohort - Clinic Cases
Matched Test Cohort
Precision
Recall
0.0
0.2
0.4
0.6
0.8
1.0

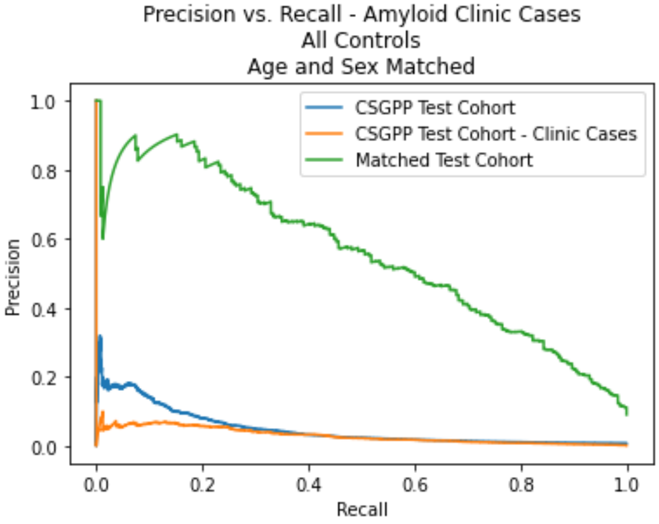
Precision vs. Recall - Amyloid Clinic Cases
All Controls
Age and Sex Matched
CSGPP Test Cohort
CSGPP Test Cohort - Clinic Cases
Matched Test Cohort
Precision
Recall
0.0
0.2
0.4
0.6
0.8
1.0

Supplemental Figures 3A and 3B

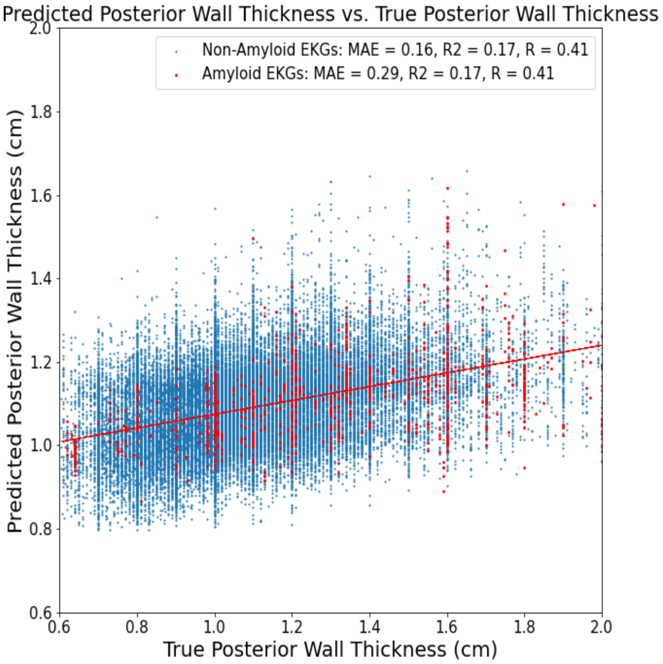

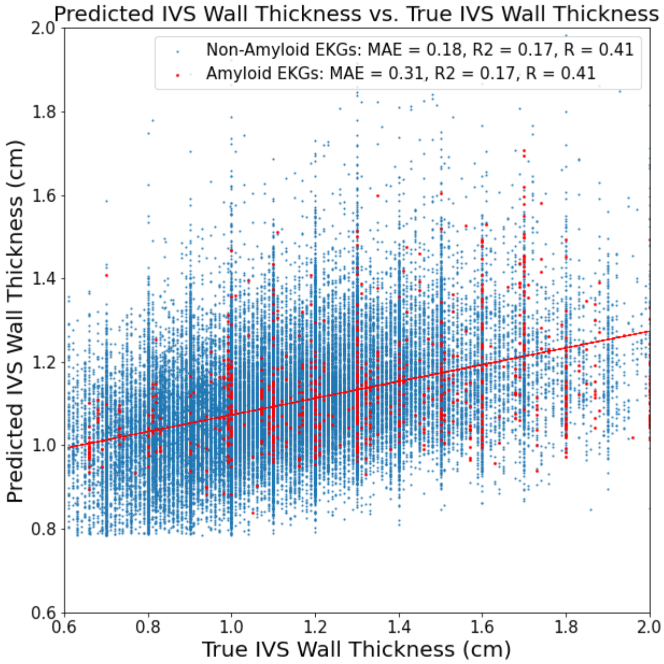

Supplemental Figure 3A – Predicted LVPW Thickness: Prediction of Left Ventricular Posterior Wall (LVPW) Thickness from ECGs using a deep learning model. Data points representing ECGs corresponding to a diagnosis of amyloidosis are marked red, while ECGs not corresponding to a diagnosis of amyloidosis are marked blue.

Supplemental Figure 3B – Predicted IVS Thickness: Prediction of interventricular septal (IVS) thickness from ECGs using a deep learning model. Prediction of Left Ventricular Posterior Wall (LVPW) Thickness from ECGs using a deep learning model. Data points representing ECGs corresponding to a diagnosis of amyloidosis are marked red, while ECGs not corresponding to a diagnosis of amyloidosis are marked blue.

Supplemental Figures 4A and 4B

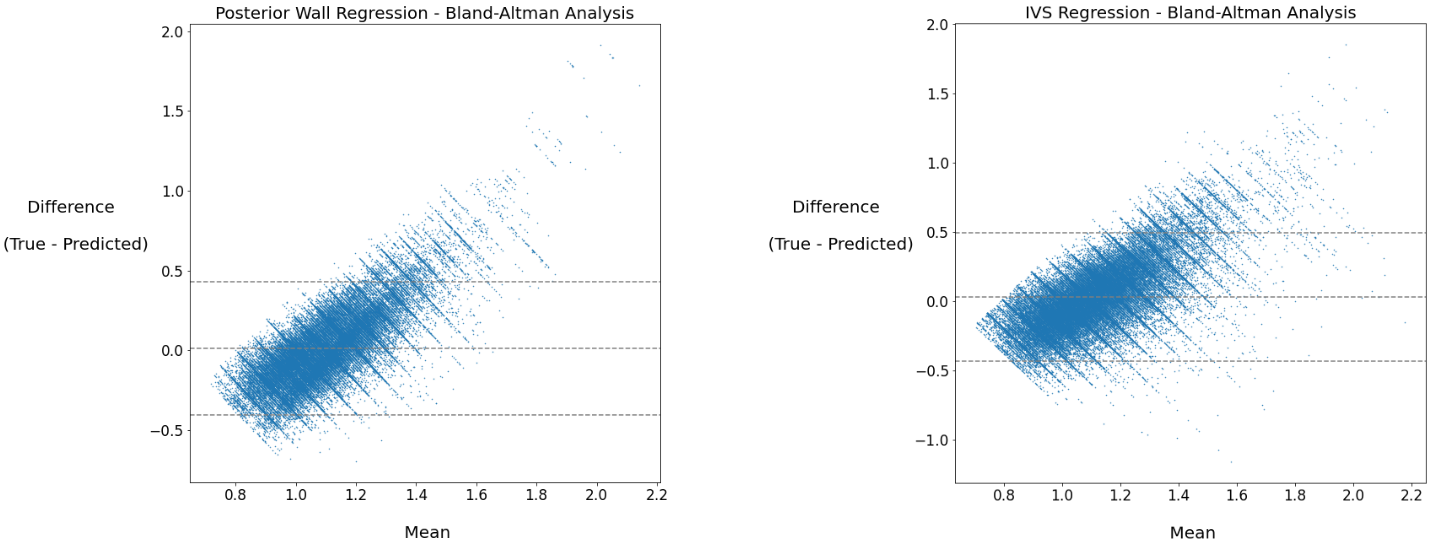

Supplemental Figure 4A – Bland-Altman plot comparing predicted wall thickness of Posterior Wall with true left ventricular posterior wall thickness. The difference between predicted and true LVPW thickness was, on average, 0.011 ± 0.210.

Supplemental Figure 4B - Bland-Altman plot comparing predicted wall thickness of the Interventricular Septum with true interventricular septal thickness. The difference between predicted and true IVS thickness was, on average, 0.031 ± 0.237.
